# Oleofoams: The impact of formulating air-in-oil systems from a lipid oxidation perspective

**DOI:** 10.1016/j.crfs.2024.100690

**Published:** 2024-01-29

**Authors:** Lucie Ribourg-Birault, Anne Meynier, Simon Vergé, Emeline Sallan, Alice Kermarrec, Xavier Falourd, Claire Berton-Carabin, Anne-Laure Fameau

**Affiliations:** a INRAE, UR BIA, F-44300, Nantes, France; b INRAE, PROBE/CALIS Research Infrastructures, BIBS Facility, F-44300, Nantes, France; c Wageningen University & Research, Laboratory of Food Process Engineering, 6700 AA, Wageningen, the Netherlands; d Univ. Lille, CNRS, INRAE, Centrale Lille, UMET, F-59000, Lille, France

**Keywords:** Oleofoam, Oleogel, Lipid oxidation, Lipid crystallization, Oleogelator

## Abstract

Air-in-oil foams, or oleofoams, have a great potential for food applications as they can at least partially replace animal or hydrogenated fats, without compromising on textural properties. Yet, there are some challenges to tackle before they can largely be implemented for real-life applications. One of those is the lack of data regarding their oxidative stability. This is an important point to consider, as although using oils rich in polyunsaturated fatty acids (PUFAs) is highly desirable from a nutritional perspective, these fatty acids are particularly prone to oxidation, which leads to major degradations of food quality. This work thus aimed to investigate the oxidative stability of oleofoams prepared with omega-3 PUFA-rich vegetable oils (rapeseed or flaxseed oil) and various types of high melting point lipid-based oleogelators (stearic acid, glyceryl monostearate and stearyl alcohol) when incubated at room temperature. The physical structure and stability of the oleofoams was monitored by various techniques (visual observations, microscopy, DSC, NMR, SAXS and WAXS). Lipid oxidation was assessed by combined measurements of primary (conjugated diene hydroperoxides) and secondary (thiobarbituric acid reactive substances – TBARS) products. We found that the oxidative stability of oleofoams was higher compared to that of the corresponding bulk oil. This protective effect was also found when the oil was simply mixed with the oleogelator without incorporation of air bubbles (i.e., forming an oleogel), and was somewhat modulated depending on the type of oleogelator. These results suggest that oleogelators and the structural changes that they induce limit the cascaded propagation of lipid oxidation in oil-continuous matrices, which is promising in the perspective of future applications.

## Highlights

• Oleogels and foams were prepared with vegetable oils and lipid-based organogelators.
• Glyceryl monostearate led to the lowest foam overrun.
• Flaxseed oleofoams could be stabilized by a stearic alcohol and acid mixture.
• The oxidative stability increased as oleogels ≥ oleofoams > oil.
• Slight differences between organogelators were noticed, except for monostearate.

## Graphical abstract

**Figure:**
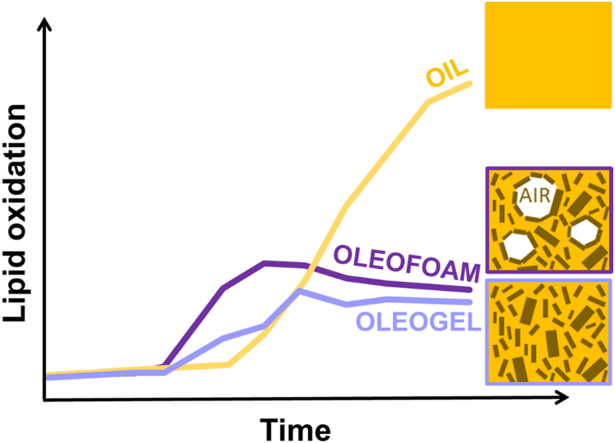

## Abbreviations

GM: Glyceryl monostearate
SAC: Stearic acid
SAL: Stearyl alcohol
GE: Oleogel
FO: Oleofoam
CD: Conjugated dienes
TBARS: Thiobarbituric acid reactive substances
NMR: Nuclear magnetic resonance
DSC: Differential scanning calorimetry
SAXS: Small angle X-ray scattering
WAXS: Wide angle X-ray scattering
SFC: Solid fat content

## 1 Introduction

Since the late 1990s′ and the first works about gelation of organic liquids using low-molecular-weight structuring agents, this research topic has attracted a lot of attention especially for the food industry, and more recently for the cosmetic and pharmaceutical industry (Terech and Weiss, 1997). For the food industry, oil structuring was traditionally achieved by the presence of high melting point triglycerides (Marangoni et al., 2012). Triglyceride crystals form a colloidal network capable of immobilizing liquid oil. This crystalline network is responsible not only for desirable organoleptic properties such as melt-in-mouth effects, but also for physical and functional properties such as texture, spreadability, hardness and crispiness (Marangoni, 2004). However, these high melting point triglycerides are often predominantly made of saturated and/or *trans*-fatty acids, which have been criticized for their nutritional impacts, in particular the *trans* ones (Oteng and Kersten, 2020; Teegala et al., 2009). Moreover some international agencies such as EFSA or WHO published some guidelines on their intake ((EFSA, 2010; WHO Guidelines, 2023; WHO Guidelines, 2023a, WHO Guidelines, 2023b). Furthermore, recent scientific reviews (Astrup et al., 2020, 2021), which recommend on the contrary not adding limitations on the consumption of saturated fats, particularly in native foods such as whole milk, cocoa, as well as a book chapter (Li, 2022). Recently, the food industry has therefore been under pressure to find alternative solutions to structure edible oils using low amount of saturated fats and eliminating industrial sources of *trans*-fatty acids (L'Abbé et al., 2009). Therefore, the use of structuring agents appears as a possible alternative to structure edible oils by decreasing the proportion of saturated fat in the final formulation of the products (Marangoni and Garti, 2018). These structuring agents are referred to as oleogelators or organogelators, and the resulting oil-based gel, as oleogel (Co and Marangoni, 2012). To be complete regarding the terminology, it should be pointed out that *stricto sensu*, oleogelation is based on oleogelators that are oil-soluble at high temperature, and induce structure (gel) formation upon cooling. Yet, oil structuring may also be achieved *via* alternative, indirect approaches including emulsion-templated, foam-templated, and solvent-exchange methods (Li et al., 2022). These methods thus require more steps compared to direct oleogelation, but open perspectives for using non-oil-soluble ingredients as structuring agents, such as proteins (Scholten, 2019). In the present work, we will focus on direct oleogelation. For this purpose, two main categories of oleogelators have been described in the literature based on their molecular weight: low molecular-weight organic gelators (LMWGs) and polymeric gelators. So far, most of the work has focused on LMWGs, mainly because most of the food-grade polymers are hydrophilic and very few can be used to produce oleogels *via* direct oleogelation (Giacintucci et al., 2018; Gravelle et al., 2017; Wang et al., 2016). The most described LMWGs are waxes, sterol-based gelators, fatty acids, fatty alcohols, and monoglycerides (Co and Marangoni, 2012; Scharfe and Flöter, 2020). They may have different gelation mechanisms resulting in different structural and macroscopic properties of the oleogel (Pernetti et al., 2007). The nature of the oil phase used also plays an important role in the final oleogel properties in terms of structure and rheological properties (Callau et al., 2021; Scharfe et al., 2021; Scharfe and Flöter, 2020). Many recent studied focused on the applications of oleogels in foods such as cakes, biscuits, sausages, and many other products as alternatives to reduce the portion of saturated or *trans* fats (Scharfe and Flöter, 2020). It is well recognized that not only the oleogel physical properties are important, but also the sensorial properties and the oxidative stability are critical parameters to take into account when using oleogels for food applications (Hwang, 2020).

In the past decade, oleofoams based on the aeration of oleogels have appeared in the literature (Binks et al., 2016; Brun et al., 2015; Callau et al., 2020a; Grossi et al., 2023; Gu et al., 2023; Metilli et al., 2021). The interest in using oleofoams for edible applications is high since they combine the oil structuring advantages of the oleogels with the presence of gas bubbles, resulting in both a reduced fat content and new appealing textures and sensorial properties (Fameau and Saint-Jalmes, 2020). However, despite the potential benefits of these oil foams for food applications, many unknowns remain to be explored (Binks and Vishal, 2021; Fameau and Binks, 2021; Heymans et al., 2017). Among the outstanding questions is the oxidative stability of these foams. Indeed, oil foams are most often formulated from vegetable oils, which may contain substantial levels of polyunsaturated fatty acids (PUFAs). PUFAs (in particular, of the n-3 series) are highly beneficial from a nutritional perspective, but they are highly sensitive to oxidation, which is a concern for the food industry. In fact, lipid oxidation leads to the appearance of defects, such as a pronounced rancid flavour even at low levels of oxidation and a deterioration of the nutritional qualities of the product (Berton-Carabin et al., 2014; Frankel, 1984; Jacobsen, 2010; Schaich, 2020a). The incorporation of air to create the foam may pose an additional risk of oxidation, but, to the best of our knowledge, this has never been documented so far. The oxidative stability of such oil-based foams is therefore a fundamental aspect that needs to be explored and understood in order to optimize their formulation and storage conditions.

**Fig. 1:**
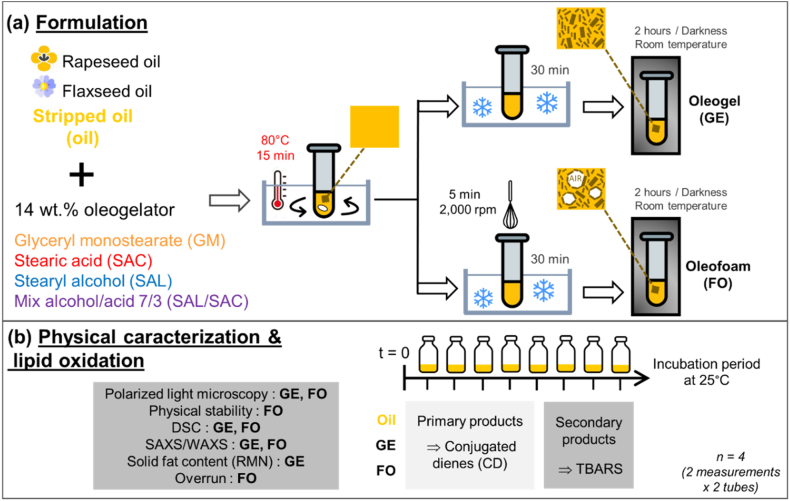
schematic representation of formulation (a) and physical characterization of oleofoams and oleogels and monitoring lipid oxidation (b).

The aim of our study was to fill this gap of knowledge by assessing the oxidative stability of model oleofoam systems, and comparing it with that of the corresponding oleogels and bulk oils. We chose two vegetable oils: (1) rapeseed oil, prominently represented in food applications, rich in unsaturated fatty acids (typically, ∼67 % oleic acid, C18:1 n-9; 16 % linoleic acid, C18:2 n-6; and 9 % α-linolenic acid (ALA), C18:3 n-3) and presenting a nutritionally balanced n-6/n-3 fatty acid ratio (EFSA, 2010; Sioen et al., 2017); and (2) flaxseed oil, one of the richest edible plant oils in ALA (∼50 %) (Dubois et al., 2007) (Fig. 1). Both oils were preliminary stripped from endogenous antioxidants to simplify the lipid oxidation pathways involved, and not to induce multiple factors varying between the different systems. We used various oleogelators that had been previously identified as suitable for oleofoam physical stabilization (Callau et al., 2020b; Fameau and Binks, 2021; Grossi et al., 2023): stearic acid, stearyl alcohol, and monoglyceride stearate, which are high melting point lipids, all of them with a chain length of 18 carbons. We also used a mixture of stearyl alcohol and stearic acid in weight ratio 7:3, known to be very efficient to produce physically stable oleofoams and oleogels in comparison to pure stearic acid and stearyl alcohol (Blach et al., 2016; Callau et al., 2020a, 2020b). We first studied the structure and physical stability over time of both oleofoams and oleogels prepared with the different oleogelators (Fig. 1). Then, we monitored the oxidation of the different systems upon storage by assessing the formation of conjugated dienes (CD), which are primary oxidation products, and the formation of thiobarbituric acid reactive substances (TBARS) among which malondialdehyde (MDA), which is a secondary oxidation product. All of these experiments were conducted by comparing the foams to oleogels, which are the intermediate step in foam formation before air is incorporated into the system. The comparison with bulk oil helped us to highlight possible inherent effects of the oleogelators on lipid oxidation.

## 2 Materials and methods

### 2.1 Materials

Vegetable oils were purchased from a local supermarket: rapeseed oil (brand U, France) and flaxseed oil (La Tourangelle, France). Solvents HPLC grade (hexane, 2-propanol, ethanol, methanol, Methyl-ter-butyl-ether: MTBE) were purchased from Biosolve (Dieuze, France). The following oleogelators were used as received: stearic acid (CAS 57-11-4, 175366, Sigma-Aldrich, Saint Quentin Falavier, France), France), stearyl alcohol (CAS 112-92-5, 0709, Sigma-Aldrich, Saint Quentin Falavier, France), and glyceryl monostearate (CAS 31566-31-1, 43883, Thermoscientific, Les Ulis, France). In the following text, for clarity, stearic acid, stearyl alcohol and glyceryl monostearate are abbreviated as SAC, SAL and GM, respectively. Hydrochloric acid (HCl) was purchased from Carlo-Erba (Val-de-Reuil, France), ethylenediamine tetraacetic acid disodium salt dihydrate (EDTA), butyl hydroxytoluene (BHT), thiobarbituric acid (TBA), trichloroacetic acid (TCA), 1,1,3,3 tetraethoxypropane (TEP) were purchased from Sigma-Aldrich (Saint Quentin Falavier, France).

Both rapeseed and flaxseed oils were first purified using aluminum oxide powder (EcoChrom™ MP Alumina N-Super I, MP Biochemicals, Germany) to remove surface-active impurities and endogenous lipophilic antioxidants, such as tocopherols and tocotrienols (Berton et al., 2011a). The efficiency of the stripping procedure was assessed by the quantification of tocopherols and tocotrienols in the purified oils as previously described (Kabri et al., 2013). Briefly, the oil was diluted to a concentration of 200 mg/mL in hexane, and injected in a liquid chromatograph (U-HPLC) paired with a silica column (Dionex advantage polar 2, 250 mm × 3 mm ID, 3 μm) and equipped with a fluorescence detector (Supplementary information, Fig. S1 and Table S1). The fatty acid composition of the purified oils and of the oleogelators in the form of free or esterified fatty acids (i.e., SAC and GM) was determined by gas chromatography after methylation in the presence of boron fluoride-methanol, according to the method described by Morrison and Smith (1964) (Supplementary information, Table S2).

### 2.2 Preparation and physical properties of oleofoams and oleogels

#### 2.2.1 Preparation of oleogels and oleofoams

We selected three different oleogelators, all three having the same alkyl chain length of 18 carbons: stearyl alcohol, stearic acid, and glyceride monostearate (abbreviated in the following as SAL, SAC and GM, respectively). We also studied the mixture of SAL and SAC with a weight ratio of 7:3, known in the literature to induce the formation of mixed crystals in high quantity, leading to a higher physical stability of the oleofoams and oleogels in comparison to the pure components, SAC and SAL (Blach et al., 2016; Callau et al., 2020a). The total oleogelator concentration was kept constant at 14 wt%, i.e., vegetable oil represented 86 wt% of the mixture. We chose this concentration based on preliminary tests in order to have enough crystals to form both oleofoams and oleogels in all the systems. Accordingly, in a 50-mL Pyrex glass tube, 1 g of oleogelator and 6 g of purified oil were introduced. The mixture was stirred with a magnetic bar for 15 min in a water bath at 80 °C to ensure the complete melting and solubilization of the oleogelator. Then, the tubes were rapidly cooled down by putting them in an iced water bath for 30 min. The oleofoam was produced while whipping the mixture for 5 min with a home-made whisk (1.8 cm diameter, 2.5 cm height) at a speed of 2000 rpm (RZR2041, HEIDOLPH) during the cooling process (Fameau et al., 2015). After whipping, the tubes were kept in the iced water bath for 30 min. The oleogels and oleofoams were then stored away from light at 20 °C until use (for the oxidative stability experiments, the waiting time did not exceed 2 h).

#### 2.2.2 Characterization of crystal shape and bubble size by polarized light microscopy

A polarized light microscope (Olympus BX51, Olympus, Japan) connected to a camera (Olympus, U-TVO.5XC-3, Olympus, Japan) was used to take photomicrographs of oleofoams and oleogels. A 10× magnification was used and images were captured digitally and analyzed with the Archimed software (Microvision Instrument, France). A small amount of sample (oleofoam or oleogel) was placed on a glass microscope slide and covered with a thin glass coverslip. Samples were observed at room temperature to visualize the shapes and sizes of the crystalline particles and air bubbles.

The bubble size was determined as described by Gaillard et al. (2015). The images were processed with the image analysis software Image J (https://imagej.net/ij/). The images were thresholded, and then the software was used to determine the mean bubble diameter.

#### 2.2.3 Molecular packing and physical organization of the lipid crystals by small- and wide-angle X-ray scattering

Small-angle (SAXS) and wide-angle X-ray scattering (WAXS) experiments were performed to determine the molecular packing inside the crystals, for oleofoams and oleogels. The diffraction patterns were monitored by recording X-ray diffraction diagrams during 10 min on a Bruker D8 Discover diffractometer (Bruker, France). Cu Kα_1_ radiation (Cu Kα_1_ = 1.5405 Å), produced in a sealed tube at 40 kV and 40 mA was selected and parallelized using a Gobël mirror optics system and collimated to produce a 500-μm diameter beam. The temperature was kept at 25 °C throughout the measurement using a HFS 91-CAP Peltier plate (Linkam). The samples were loaded in a specific cell for gels.

#### 2.2.4 Solid fat content

The solid fat content (SFC) for all oleogels was determined using a 20 MHz (0.47 T) mq20 Series Bruker pulse NMR Spectrometer (Bruker, France). The oleogels were molten at 80 °C, then poured into 10-mm diameter glass NMR tubes. Then, they were left to crystallize at 25 °C for 24 h before measurement. The tubes containing the oleogels were placed into the NMR spectrometer and the temperature of the ^1^H probe was thermostated with a precision of 0.1 K. Temperature was increased stepwise from 20 °C to 50 °C in five degrees increments. The oleogel was equilibrated for 30 min at each temperature before measurement. Free induction decay (FID) signal was acquired with a dwell time of 1 μs, using a relaxation delay of 10 s and 128 scans. The parameters of the AOCS method (AOCS Official Merthod, 1999) were adjusted in particular for the recycling time. Indeed, a value of less than 10 s led to underestimating the SFC. (Supplementary Information Table S3). This result can be explained by the fact that the solid parts have very long T_1_ relaxation times, since the autocorrelation time τ_c_ value is high (Besghini et al., 2019). Each kinetic of SFC in function of temperature was conducted in triplicate. The SFC was determined using the method proposed by Linde & al (Linke et al., 2018). Briefly, the intercepts of the fast and slowly decaying parts of the curve are respectively associated with solid and liquid part of the signal. The SFC was calculated according to Equation (1):

$$SFC=\frac{I_{S+L}-I_{L}}{I_{S+L}}\times 100$$

where I_S + L_ is the intercept of the total signal and I_L_ is the intercept of the liquid signal.

In order to compare NMR results with other compositional data, SFC was normalized according to the molar mass and the proton density of each oleogel.

For technical reasons, this analysis could not be conducted on oleofoams, as they could not be loaded into the NMR tubes without foam physical destruction.

#### 2.2.5 Thermal behavior of oleofoams and oleogels

The thermal properties of oleofoams and oleogels were assessed by differential scanning calorimetry (DSC) (TA Q200 instrument, New Castle, UK). Around 20 mg of the sample were placed into a hermetically sealed aluminium pan for testing. An empty aluminium pan was used as a reference. DSC runs were carried out from 10 °C to 80 °C. The samples were scanned at a constant rate of 2 °C.min^−1^ applying a heating-cooling cycle (two heating steps, with one cooling step in between). An isotherm of 10 min was applied at 80 °C before cooling, and at 10 °C before heating. The transition temperatures, enthalpies of melting and crystallization were determined from the DSC curves using TA Instruments’ Universal Analysis Software, in triplicate for each sample.

#### 2.2.6 Overrun of oleofoams

To determine the overrun of oleofoams, a small cylindrical container of well-defined volume was weighed empty, and then was weighed again when filled with oleofoam. The difference corresponds to the mass of oleofoam. The oleofoam was then melted and weighed again as described by Brun et al. (2015). The difference corresponds to the mass of melted oleofoam.

The overrun was defined as the volume percentage of air incorporated into the oil-based melted oleofoam (Equation (2)):

(Equation 2) Overrun (%) = ((m _remelted oleofoam_ – m _oleofoam_) / m _remelted oleofoam_) x 100

The test was performed in triplicate.

### 2.3 Lipid oxidation

#### 2.3.1 Incubation conditions

For each experiment series, 12 aliquots of 1.5 g of sample (oil, oleofoam or oleogel) were placed in 22.4-mL headspace tubes, hermetically sealed and stored in an oven, in the dark, at 25 °C. At each time point, 2 tubes were taken out of the oven, and 2 measurements were performed per tube. For rapeseed oil-based samples, the incubation was conducted over 72 days, with measurements of the conjugated dienes approximately every 10 days, and of the TBARS after 0, 36 and 72 days. For flaxseed oil-based samples, the incubation was conducted over 15 days, with measurements of the conjugated dienes approximately every 4 days, and of the TBARS after 0, 8 and 15 days.

#### 2.3.2 Measurement of lipid oxidation products

Conjugated dienes (CD) were assessed as primary lipid oxidation products. They were quantified by spectrophotometry according to a previously described method (Lethuaut et al., 2002). In brief, 25 mg oil, oleofoam or oleogel were weighed into a 2-mL centrifuge tube, then melted for a few seconds in a 70 °C water bath (except for bulk oil samples). 2-Propanol was added through dilution series to obtain a final concentration of 0.25 mg lipid per mL. The absorbance spectrum was recorded between 200 and 310 nm using 2-propanol as a blank. The concentration of conjugated dienes ([CD], mmol kg^−1^ oil) was expressed as follows (Equation (3)):

(Equation 3) $$\left[CD\right]=\frac{A_{233}-A_{310}\;}{C_{lipids}\times 10^{-3}\times \varepsilon \times l}\times 1000$$

Where *A*_233_ and *A*_310_ are the absorbances of the sample at 233 nm and 310 nm, respectively; *C*_*lipids*_ is the lipid concentration in the sample measured (i.e., the final dilution in 2-propanol, in mg mL^−1^); *ε* is the molar extinction coefficient of conjugated dienes (27000 M^−1^ cm^−1^); and *l* is the optical path length (1 cm).

Thiobarbituric acid reactive substances (TBARS) were measured as a marker of secondary lipid oxidation products according to a previously described method (Viau et al., 2016). In brief, 100 mg oil, oleofoam or oleogel, melted for a few seconds in a 70 °C water bath (except for bulk oil samples), were added with 40 μL BHT solution (4.5 mM in ethanol), 80 μL EDTA solution (100 mM in water) and 10 mL TCA solution (0.3 M in water). The tubes were vortexed for 1 min and then sonicated in an ice bath for 5 min. An external calibration was conducted using dilutions from a stock solution of MDA prepared by acid hydrolysis of 1 mg/mL TEP solution in HCl 0.1 N held at 100 °C for 5 min. One milliliter of TBA reagent (55 mM) was added to 1 mL sample solution or calibration solution, after which the tubes were vortexed and placed at 70 °C for 20 min, and finally cooled down in a cold-water bath. The absorbance spectrum of the resulting medium was recorded between 450 and 650 nm. The absorbance at 600 nm was subtracted from that at 532 nm to compensate for the turbidity of the sample. The TBARS content was expressed as mmol MDA equivalent per gram of vegetable oil.

For both lipid oxidation markers, it should be pointed out that the results were expressed per gram of vegetable oil, i.e. by excluding the 14 wt% of the lipid phase corresponding to the oleogelators which, due to their composition in saturated fatty acids only, are not susceptible to oxidation.

### 2.4 Statistical analysis

Data were reported as means ± standard deviation. Variance analysis and comparison of means (Tukey HSD) were analyzed with the software XLSTAT (Version, 2023; Addinsoft, Paris, France) Threshold values of *p < 0.05* were applied. Homogeneous groups were identified by different small letters on the graphs or tables. The factors were the oleogelator type (GM, SAC, SAL, SAL/SAC) and the oil nature (rapeseed or flaxseed oil) and the following variables: overrun, melting temperature, crystallization temperature, melting enthalpy, and crystallization enthalpy.

## 3 Results and discussion

Before comparing the oxidizability of the oleofoams and oleogels, it was crucial to assess the physical stability and structure changes upon incubation period (several weeks in our case), by a multiscale approach coupling different techniques. The systems based on the different oleogelators were characterized systematically with rapeseed oil. For flaxseed oil, the incorporation of air bubbles turned out to be more difficult, therefore, this oil was used only in combination with the SAL/SAC mixture.

### 3.1 Properties and physical stability of oleofoams and oleogels

#### 3.1.1 Physical appearance and microstructure of oleofoams and oleogels

**Fig. 2:**
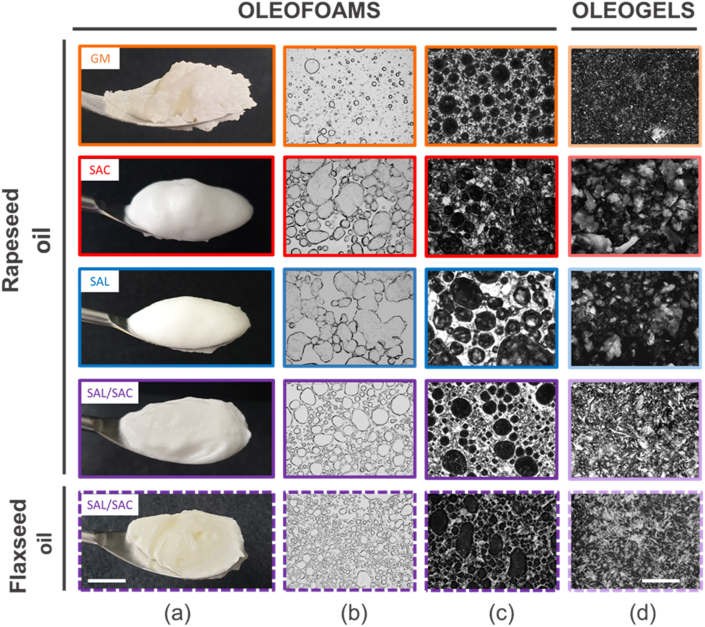
Macroscopic observation of oleofoams (a), optical microscopy images of oleofoams (b), polarized light microscopy images of oleofoams (c) and polarized light microscopy images of oleogels (d) in rapeseed oil-based oleofoams or in the corresponding oleogels prepared with glyceryl monostearate (GM, orange), stearic acid (SAC, red), stearyl alcohol (SAL, blue), or stearyl alcohol/stearic acid (SAL/SAC) in a weight ratio of 7:3 (purple). The dashed line corresponds to flaxseed oil-based oleofoam or in the corresponding oleogel prepared with stearyl alcohol/stearic acid (SAL/SAC) in a weight ratio of 7:3 (purple). The scale bar represents 1 cm for (a) and 500 μm for (b–d). (For interpretation of the references to colour in this figure legend, the reader is referred to the Web version of this article.)

We first tried to produce foams by whipping the oil and the oleogelator mixtures in their molten state at 80 °C for several minutes. In these conditions, it was impossible to entrap air bubbles for the systems based on SAC, SAL and on the SAL/SAC mixture (Supplementary information, Fig. S2). However, a few air bubbles could be produced and stabilized when GM was used (Supplementary information, Fig. S2). This result shows that SAL and SAC molecules were unable to stabilize the air-oil interface, whereas the GM molecules are somewhat surface-active. This result could be tentatively attributed to the possible formation of hydrogen bonds between GM and oil molecules (triacylglycerols), which is not the case for SAC and SAL (Liu and Binks, 2021). Conversely, oleofoams were easily produced by whipping during cooling of the systems, showing that oleofoam formation and stabilization mechanisms are temperature-dependent, and most likely, related to crystallization events in the matrices. Qualitatively, by naked-eye observation, the appearance of the oleofoams was different depending on the oleogelator used: liquid-like foams for SAC and SAL, and gelified oleofoams for GM and SAL/SAC mixture (Fig. 2a). By using polarized light microscopy, we observed that for all the systems, the air bubbles were stabilized by crystals, which not only surround the air bubbles but were also present in large amounts between the bubbles in the oily phase (Fig. 2 b and c). The GM-based oleofoam exhibited fewer and smaller bubbles (mean bubble diameter 32 ± 26 μm) than the oleofoams based on SAC and SAL (mean bubble diameter equal to 154 ± 65 μm and 177 ± 58 μm, respectively). The oleofoam based on the SAL/SAC mixture showed bubbles in high quantity and with a small size (mean bubble diameter 71 ± 42 μm) in comparison to the oleofoams based on the pure components (SAC and SAL). The macroscopic appearance and the microscopic pictures were fairly similar for the oleofoams based on rapeseed and flaxseed oils. We suppose that for the oleofoams based on SAC, SAL and on the mixture of SAL/SAC, the arrangement of the crystals at the interface occurs with their faces composed of low energy methyl (-CH_3_) groups in contact with air. The edges expose mainly methylene and carboxylic or hydroxyl groups which interact with each other at the air-oil surface (Fameau and Binks, 2021). In the case of the GM-based oleofoams, some of the GM molecules may already stabilize the air bubbles before the onset of crystallization, and the cooling step associated with crystal formation helped to stabilize the oleofoams due to both interfacial and bulk GM crystallization (Liu and Binks, 2021).

As reference systems, we also assessed the structure and properties of oleogels of similar composition to that of the respective oleofoams, but without the incorporation of air upon cooling. Just after preparation at 80 °C, all the systems were liquid and flowed when the tubes were turned over. Upon rapid cooling, they all transited into gels, as exemplified by the fact that all the samples could be turned upside down without flowing. The microstructure of the oleogels was studied by polarized light microscopy (Fig. 2d). For the oleogel based on GM, small needle-like structures were observed as already described in the literature (Fig. 2d) (Ferro et al., 2019). For pure SAL and SAC, we observed large platelet-like crystals (Fig. 2d). The length and the width of the crystals were in the order of magnitude of around 100 μm. These images and the associated structures look similar to the ones described in the literature for SAL and SAC oleogels based on other vegetable oils (Blach et al., 2016; Callau et al., 2020a; Gravelle et al., 2017). For the SAL/SAC mixture, smaller platelet-like crystals were observed, below 50 μm, which was in accordance with the literature (Fig. 2d) (Blach et al., 2016; Schaink et al., 2007). The crystals looked very similar in both oils (rapeseed and flaxseed).

**Fig. 3:**
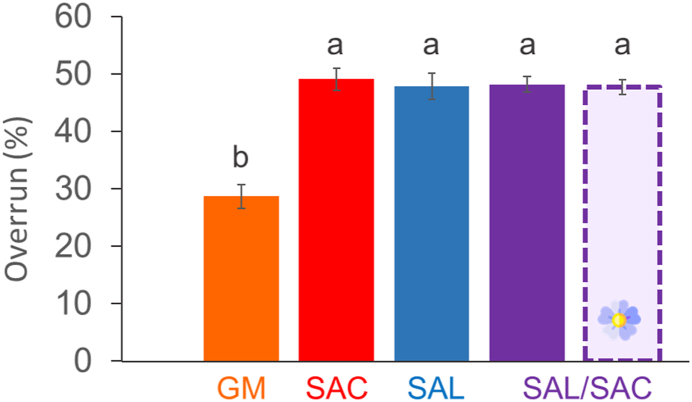
Overrun (%) of rapeseed oil-based oleofoams prepared with glyceryl monostearate (GM, orange), stearic acid (SAC, red), stearyl alcohol (SAL, blue), or stearyl alcohol/stearic acid (SAL/SAC) in a weight ratio of 7:3 (purple). The last one (purple dots) corresponds to flaxseed oil-based oleofoam prepared with stearyl alcohol/stearic acid (SAL/SAC) in a weight ratio of 7:3. The error bars correspond to the standard deviation (n = 3) and the small letters corresponds to the statistically homogeneous groups. (For interpretation of the references to colour in this figure legend, the reader is referred to the Web version of this article.)

To complete these qualitative macroscopic and microscopic observations of the oleofoams, we determined the overrun for all the systems (Fig. 3). The lowest overrun was obtained for the GM foam (28.6 ± 2.0 %). The overruns for all the other systems were not significantly different (*p > 0.05*): 49.1 ± 1.9 %, 47.9 ± 2.3 %, 48.1 ± 1.4 % and 47.6 ± 1.3 %, for SAC, SAL, SAL/SAC in rapeseed oil and SAL/SAC in flaxseed oil, respectively. The values of the overrun were in accordance with the optical microscopy pictures showing less bubbles for the GM oleofoam than for the other oleofoams. No effect of the type of oil in the case of the SAL/SAC mixture was observed. Moreover, these values were similar to the overruns obtained in previous work for SAL, SAC and SAL/SAC systems, yet with non-purified oils, with the same foaming protocol (Callau et al., 2020).

**Fig. 4:**
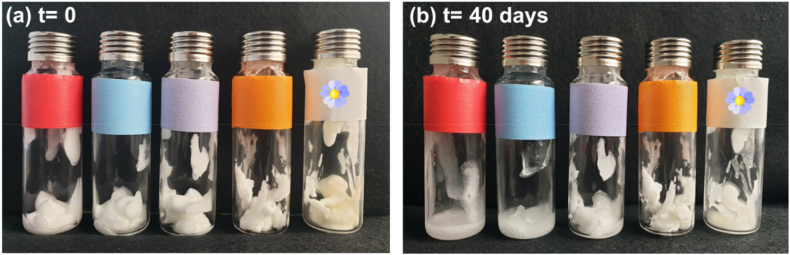
Photographs of headspace tubes containing 1.5 g of rapeseed oil-based oleofoams prepared with glyceryl monostearate (GM, orange), stearic acid (SAC, red), stearyl alcohol (SAL, blue), or stearyl alcohol/stearic acid (SAL/SAC) in a weight ratio of 7:3 (purple) on day 0 (A) and after 40 days at 25 °C in the darkness. The last tube (white tape) corresponds to flaxseed oil-based oleofoam prepared with stearyl alcohol/stearic acid (SAL/SAC) in a weight ratio of 7:3. (For interpretation of the references to colour in this figure legend, the reader is referred to the Web version of this article.)

The physical stability of oleofoams was followed by visual inspection over storage at 25 °C directly in the headspace tubes used for the lipid oxidation measurements (Fig. 4). Upon storage, drained liquid oil was observed after 40 days for oleofoams prepared with rapeseed oil and pure SAC and SAL. Drainage was reduced for oleofoams prepared with rapeseed oil and SAL/SAC mixture. This result is in agreement with a previous study showing a better stabilization of oleofoams by SAL/SAC mixtures than by the pure components alone (Callau et al., 2020). No liquid oil drainage was observed for the oleofoams prepared with rapeseed oil and GM. In the same way, the oleofoam based on flaxseed oil only showed a slight drainage with the SAL/SAC mixture, suggesting no effect of the type of oil used.

#### 3.1.2 Thermal behavior of oleofoams and oleogels

**Fig. 5:**
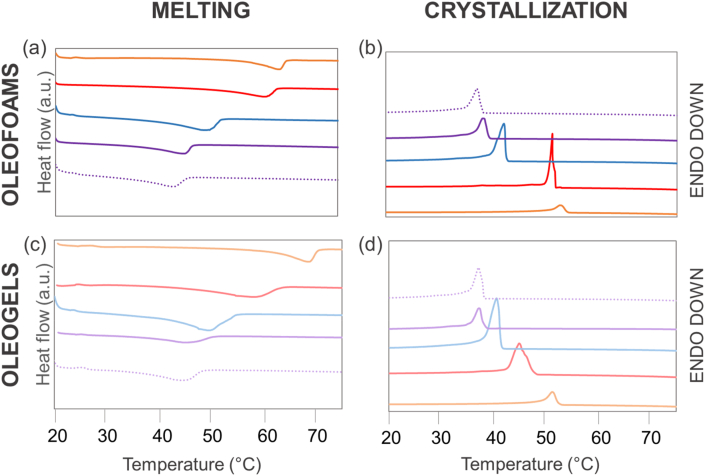
Evolution of the peak temperatures for melting (a and c) and crystallization (b and d) of rapeseed oil-based oleofoams (top) or in the corresponding oleogels (bottom) prepared with glyceryl monostearate (GM, orange), stearic acid (SAC, red), stearyl alcohol (SAL, blue), or stearyl alcohol/stearic acid (SAL/SAC) in a weight ratio of 7:3 (purple). The dotted curve corresponds to flaxseed oil-based oleofoam or in the corresponding oleogel prepared with stearyl alcohol/stearic acid (SAL/SAC) in a weight ratio of 7:3 (purple). The spectra were shifted in intensity for clarity. (For interpretation of the references to colour in this figure legend, the reader is referred to the Web version of this article.)

The melting and crystallization temperatures of the high melting point lipids are important parameters when it comes to the properties of oleofoams (Fameau and Binks, 2021), in particular their stability upon storage at various temperatures. The thermal behavior of the oleofoams and oleogels was studied by DSC (Fig. 5). All the systems exhibited a wide endothermic peak upon melting and a wide exothermic peak upon crystallization. The melting and crystallization temperatures with the associated enthalpies are shown in Table 1. We observed that all the melting temperatures were slightly higher for the oleofoams than for the oleogels. We suppose that it may be due to a limited yet existing drainage of oil at the bottom of the foam samples, which would result in a higher relative fraction of high melting point components – the oleogelators – in the top part of the foam samples, where sampling was performed for subsequent DSC analysis. The GM-based oleofoam exhibited the highest melting and crystallization temperatures with a melting temperature of 64.1 ± 0.5 °C and a crystallization peak at 54.3 ± 0.1 °C, but the lowest crystallization and melting enthalpy. The SAC-based oleofoam exhibited a melting temperature of 59.2 ± 0.4 °C and a crystallization peak at 51.5 ± 0.4 °C, whereas the SAL-based oleofoams exhibited lower melting and crystallization temperatures (47.9 ± 0.9 °C and 42.6 ± 0.3 °C, respectively). The oleofoams based on the SAL/SAC mixtures exhibited the lowest melting and crystallization temperatures (43.8 ± 0.1 °C and 38.0 ± 0.4 °C, respectively). Similar values were obtained for the oleofoam based on the SAL/SAC mixture and flaxseed oil (43.8 ± 0.1 °C and 38.0 ± 0.4 °C, for the melting and the crystallization temperatures, respectively). This is consistent with the previously reported lack of an effect of the oil type (rapeseed vs flaxseed) for this oleogelator system. The trend observed for the oleofoams was thus the same as the one observed for the oleogels (Table 1). Besides, the lower melting and crystallization temperatures for the SAL/SAC mixtures in comparison to the pure components SAC and SAL are in accordance with the literature (Blach et al., 2016).

**Table 1:** Melting and crystallization temperature and enthalpy of rapeseed oil-based oleofoams or in the corresponding oleogels prepared with glyceryl monostearate (GM, dark/light orange), stearic acid (SAC, dark/light red), stearyl alcohol (SAL, dark/light blue), or stearyl alcohol/stearic acid (SAL/SAC) in a weight ratio of 7:3 (dark/light purple). The line named SAL/SAC with flaxseed flower corresponds to flaxseed oil-based oleofoam or in the corresponding oleogel prepared with stearyl alcohol/stearic acid (SAL/SAC) in a weight ratio of 7:3 (dark/light purple). Results are presented as mean ± SD of three independent measurements.

	oleogelator	Melting temperature (Tm, °C)	Crystallization temperature (Tc, °C)	Melting enthalpy (Δm, J/g)	Crystallization enthalpy (Dc, J/g)
**OLEOFOAMS**	GM	64.1 ± 0.5	54.3 ± 0.1	15.1 ± 0.6	10.0 ± 0.8
	SAC	59.2 ± 0.4	51.5 ± 0.4	35.4 ± 3.9	37.1 ± 3.7
	SAL	47.9 ± 0.9	42.6 ± 0.3	36.5 ± 11.7	40.5 ± 11.9
	SAL/SAC	43.8 ± 0.1	38.0 ± 0.4	25.2 ± 1.9	30.2 ± 0.6
	SAL/SAC (flax)	42.7 ± 0.5	37.1 ± 0.1	18.8 ± 0.5	22.2 ± 0.5

**OLEOGELS**	GM	64.1 ± 0.2	51.5 ± 0.2	14.8 ± 6.7	12.7 ± 1.2
	SAC	55.2 ± 1.4	47.0 ± 2.8	27.7 ± 4.9	28.2 ± 6.5
	SAL	47.4 ± 0.4	40.6 ± 0.5	35.2 ± 7.6	38.8 ± 7.1
	SAL/SAC	43.4 ± 0.1	37.2 ± 0.2	27.4 ± 1.4	29.4 ± 0.9
	SAL/SAC (flax)	42.2 ± 0.2	37.2 ± 0.4	27.5 ± 0.7	27.6 ± 2.1

#### 3.1.3 Solid fat content of the oleogels

**Fig. 6:**
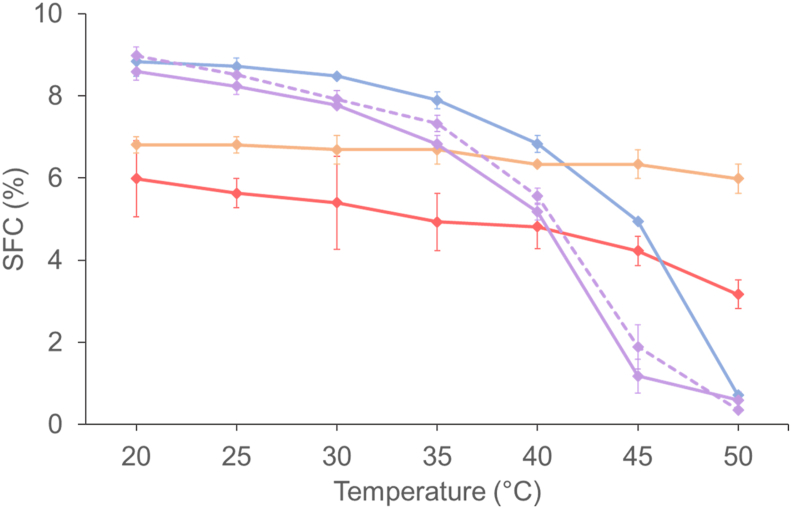
Solid fat content (SFC) profiles of rapeseed oil-based oleogels prepared with glyceryl monostearate (GM, orange), stearic acid (SAC, red), stearyl alcohol (SAL, blue), or stearyl alcohol/stearic acid (SAL/SAC) in a weight ratio of 7:3 (purple). The dotted curve corresponds to flaxseed oil-based oleogel prepared with stearyl alcohol/stearic acid (SAL/SAC) in a weight ratio of 7:3 (purple). (For interpretation of the references to colour in this figure legend, the reader is referred to the Web version of this article.)

The quantity of crystals is a key parameter governing the foaming properties in terms of overrun and stability (Fameau and Binks, 2021). The quantity of crystals is linked to the SFC, and it can be determined by NMR. However, this characterization method could be conducted only on oleogels, as it is technically not possible to fill in the NMR capillaries with oleofoams. In Fig. 6, the evolution of the solid fat content (SFC) for all oleogels as a function of temperature is shown. The overall melting profiles from 20 °C to 50 °C looked fairly similar for the oleogels based on SAL and on the mixture SAL/SAC, showing the same melting behavior only slightly shifted to lower SFC by using the mixture in comparison to pure SAL. They exhibited a similar profile with a sharp decrease in SFC from 35 °C onwards. For the SAC-based oleogel, the SFC was low and almost constant from 20 °C to 40 °C (from 6 to 5 %), and then began to decrease. For the GM-based oleogel, the SFC remained almost constant (from 7 to 6 %) when rising the temperature from 20 °C to 50 °C. At 20 °C, the lowest SFC was around 6 % for the SAC-based oleogel. Since the mass fraction of SAC in the system was 14 wt%, this indicates that less than half of SAC was actually crystallized, and thus the remaining SAC molecules were solubilized in the rapeseed oil. For the GM-based oleogel, the SFC was higher, around 6.8 %. The highest SFC was obtained for SAL, around 8.6 %, showing that the solubility of stearyl alcohol was much lower than that of stearic acid in rapeseed oil. This result indicates that about two thirds of SAL were crystallized, whereas one third was solubilized in the rapeseed oil. By adding a limited amount of SAC in the oleogels based on SAL (weight ratio 7:3), the SFC decreased slightly to a value around 8.2 %. The SFC obtained with SAL was shown to be higher in purified oil than in non-purified oils, as described in previous work (Blach et al., 2016; Gravelle et al., 2017). By removing the minor polar components, it is therefore possible that we change the solubility of SAL in the rapeseed oil, as already described in the literature for waxes in vegetable oils (Scharfe et al., 2022). Moreover, the oleogels based on flaxseed oil with the SAL/SAC mixture exhibited the same SFC profile as for rapeseed oil, showing that the nature of the oils did not affect the SFC of the SAL/SAC mixture.

#### 3.1.4 Determination of crystal structures in oleofoams and oleogels by SAXS and WAXS

**Fig. 7:**
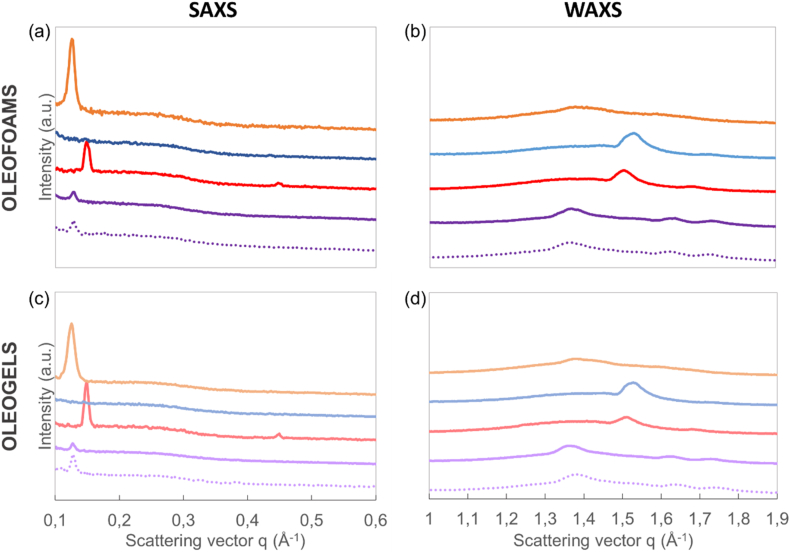
SAXS (a and c) and WAXS (b and d) spectra at 25 °C of rapeseed oil-based oleofoams (top) or in the corresponding oleogels (bottom) prepared with glyceryl monostearate (GM, orange), stearic acid (SAC, red), stearyl alcohol (SAL, blue), or stearyl alcohol/stearic acid (SAL/SAC) in a weight ratio of 7:3 (purple). The dotted curve corresponds to flaxseed oil-based oleofoam or in the corresponding oleogel prepared with stearyl alcohol/stearic acid (SAL/SAC) in a weight ratio of 7:3 (purple). The spectra were shifted in intensity for clarity. (For interpretation of the references to colour in this figure legend, the reader is referred to the Web version of this article.)

The characterization of the crystal structure within foams and gels was performed by small and wide-angle X-ray scattering (SAXS and WAXS, respectively). Both the SAXS and WAXS region are shown in Fig. 7. Table 2 shows the values for *d*-spacings for all the oleofoams and oleogels. The crystal structures found in the oleofoam systems were compared with those found in the corresponding oleogels to assess the impact of whipping on the crystal structure (Fig. 7, and Table 2).

**Table 2:** SAXS and WAXS value for *d*-spacings measured for rapeseed oil-based oleofoams or in the corresponding oleogels prepared with glyceryl monostearate (GM, dark/light orange), stearic acid (SAC, dark/light red), stearyl alcohol (SAL, dark/light blue), or stearyl alcohol/stearic acid (SAL/SAC) in a weight ratio of 7:3 (dark/light purple). The line named SAL/SAC with flaxseed flower corresponds to flaxseed oil-based oleofoam or in the corresponding oleogel prepared with stearyl alcohol/stearic acid (SAL/SAC) in a weight ratio of 7:3 (dark/light purple).

	oleogelator	SAXS d-spacing (Å)	WAXS d-spacing (Å)
**OLEOFOAMS**	GM	50.2	4.5
	SAC	41.9; 13.9	4.2; 3.7
	SAL	Not detected	4.1; 3.6
	SAL/SAC	48.3	4.5; 3.9; 3.6
	SAL/SAC (flax)	48.3	4.5; 3.9; 3.6
**OLEOGELS**	GM	50.2	4.5
	SAC	41.9; 13.9	4.2; 3.7
	SAL	Not detected	4.1; 3.6
	SAL/SAC	48.3	4.5; 3.9; 3.6
	SAL/SAC (flax)	48.3	4.5; 3.9; 3.6

In the SAXS region at 25 °C, one peak was observed for GM-based oleofoam (Fig. 7a). The peak corresponded to a *d*-spacing of 50.2 Å. In the WAXS region at 25 °C, one peak was identified (Table 2). For pure SAC-based oleofoams, in the SAXS region two main peaks were identified with the first one corresponding to *d*-spacing of 41.9 Å and the second peak corresponded to the third order reflection peak (Fig. 7). In the WAXS region, two peaks were identified giving *d*-spacing of around 4.2 and 3.7 Å. Thus, we suppose that in the SAC oleofoams, SAC crystallized in a double layer structure associated with a possible β-form (Blach et al., 2016; Fameau and Marangoni, 2022). For the SAL oleofoams, no peak was clearly distinguished in our experimental conditions in the SAXS region. In the WAXS region, two peaks were clearly identified corresponding to *d*-spacings of 4.1 and 3.6 Å. We suppose based on the literature that SAL crystallized in a double layer structure associated with a possible β-form (Blach et al., 2016; Fameau and Marangoni, 2022). For the oleofoams based on the SAL/SAC mixture, one main peak was observed in the SAXS region corresponded to a *d*-spacing of 48.3 Å, and three peaks in the WAXS region (*d*-spacings of 4.5, 3.9 and 3.6 Å). This *d*-spacing could not be associated to the *d*-spacings measured for oleofoams containing only SAL or SAC, which highlights the formation of mixed crystals of SAL and SAC as already described in the literature (Blach et al., 2016). Therefore, both components co-crystallized. Moreover, these mixed crystals showed a different polymorphic form in the WAXS region as compared to the pure SAL and SAC. The oleofoams based on flaxseed oil with the SAL/SAC mixture exhibited the same SAXS/WAXS patterns than for the rapeseed oil, showing that the same crystalline structure of mixed crystals was present for both oils. Moreover, the peak position in the SAXS and WAXS regime were very similar between the oleogels and the resulting oleofoams for all the systems showing no effect of the whipping on the crystals structure.

### 3.2 Lipid oxidation in oleofoams and oleogels

#### 3.2.1 Lipid oxidation in oleofoams

**Fig. 8:**
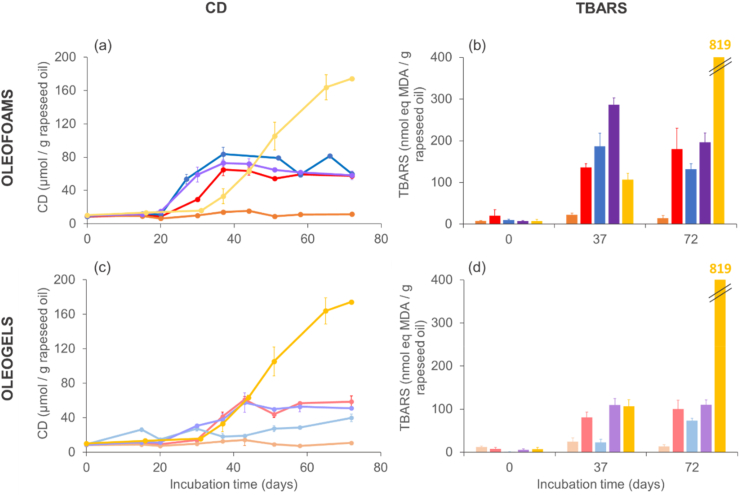
Formation of lipid oxidation products (conjugated dienes (CD), left panels; and TBARS, right panels) in rapeseed oil-based oleofoams (a, b) or in the corresponding oleogels (c, d) prepared with glyceryl monostearate (GM, orange), stearyl alcohol (SAL, blue), stearic acid (SAC, red) or stearyl alcohol/stearic acid (SAL/SAC) in a weight ratio of 7:3 (purple). The yellow symbols/bars correspond to measurements done on pure stripped rapeseed oil (no oleogelator) as a control. Error bars correspond to the standard deviations (n = 4), some of them being within the markers. (For interpretation of the references to colour in this figure legend, the reader is referred to the Web version of this article.)

To address the question of the oxidative stability of the formulated systems, we monitored the formation of primary (conjugated dienes, CD) and secondary (TBARS) lipid oxidation products upon incubation at 25 °C. As a control, the oxidation course of the corresponding bulk stripped oils (rapeseed and flaxseed oils) was also determined in the same conditions. For rapeseed oil-based samples, we assessed the effect of the type of oleogelator (Fig. 8). For all systems prepared with stripped rapeseed oil, no CD were detected for the first 20 days of incubation. After this long lag phase, their formation started to increase, with different rates and patterns according to the type of oleogelator: CD formation was quite fast in the oleofoams based on SAL, SAC and on the SAL/SAL mixture, reaching around 65–80 μmol CD/g oil after 37 days, after which it levelled off and remained at values around 75 μmol CD/g oil until the end of the incubation period. Conversely, for the GM-based oleofoam, the CD level did not show any substantial variation along the whole incubation period, suggesting that this system is highly stable to oxidation. When stripped rapeseed oil with no oleogelator was incubated in the same conditions, almost no CD formation was observed for the first 31 days, after which the formation of CD increased steadily, reaching 174 μmol CD/g oil at the end of the incubation. This value corresponds to a high level of oxidation for rapeseed oil (Berton et al., 2011b; Viau et al., 2016), and is largely higher than that observed for any of the oleofoams. As most CDs arising from lipid oxidation are supposedly found in hydroperoxide molecules, it is also interesting to compare this value with the maximum amount of hydroperoxides that could theoretically form in our samples, based on the available reactants (i.e., oxygen and oil; see calculation in Supplementary information, Table S4). Accordingly, if all of the oxygen present in the headspace tube was consumed through lipid oxidation, this would give rise to a hydroperoxide content of around 140 μmol/g oil. The fact that this value is lower than the final CD content actually measured for the bulk rapeseed oil may imply that some air leakage occurred into the tubes (and if so, in a reproducible manner, since 4 different tubes were taken per time point), or that molecules which are not hydroperoxides, yet contain CD structures were formed upon oxidation (Schaich et al., 2017). The evolution of TBARS in the various systems (Fig. 8b) was well in line with the trends observed for CDs: values ranging from 135 to 285 nmol eq. MDA/g oil were found for the oleofoams based on SAL, SAC and on the SAL/SAC mixture after 37 days of incubation, and those values did not increase further after 37 days. The GM-based foam exhibited very low TBARS values around 15–20 nmol eq. MDA/g oil along the whole incubation period. In the bulk stripped rapeseed oil, the TBARS content was in the same range as that in the oleofoams (excluding the highly stable GM-based system) after 37 days, but reached a very high value (about 820 nmol eq. MDA/g oil) after 72 days. These results suggest that oleofoams are overall more stable to oxidation in late stages of oxidation (or storage) than bulk oils, even though oleogelators are not all equally potent at achieving this protective effect. For instance, GM led to foams with very high stability, whereas the other oleogelators (SAL, SAC, and the SAL/SAC mixture) led to foams that were subjected to an onset of oxidation which occurred at an earlier stage compared to the bulk oil. Nevertheless, with those three different oleogelator systems, the same effect of levelling off the formation of lipid oxidation products (both CDs and TBARS) at a certain stage was observed, with no further evolution upon storage.

#### 3.2.2 Lipid oxidation in oleogels

To probe further the potential mechanisms involved, we followed lipid oxidation in rapeseed oil-based oleogels, i.e., systems having the exact same composition as the oleofoams, but no dispersed air phase (Fig. 8c and d). When looking at the CD formation in these systems (Fig. 8c), we also observed no or very limited oxidation until 31 days of incubation, after which CD started forming in a very similar course as in bulk oil for the SAC and SAL/SAC mixture-based oleogels. Yet, for these two systems, the formation of CD stopped proceeding after 44 days and remained at a steady level (around 55 μmol CD/g oil) thereafter. For the SAL only-based oleogel, CD formation was more limited and increased only very slowly upon incubation to reach around 40 μmol CD/g oil after 72 days. Finally, no noticeable CD formation occurred in the GM-based oleogel along the whole incubation period, which is well in line with the outstanding oxidative stability of the corresponding oleofoam. The TBARS values measured in the oleogels were consistent with the CD results, showing a very small content for the GM-based oleogel along the whole incubation. The contents were between 20 and 110 nmol eq. MDA/g oil at day 37, which did not evolve much subsequently, in opposition with the high TBARS formation observed in the bulk rapeseed oil (Fig. 8d).

#### 3.2.3 Lipid oxidation of oleofoam based on flaxseed oil

**Fig. 9:**
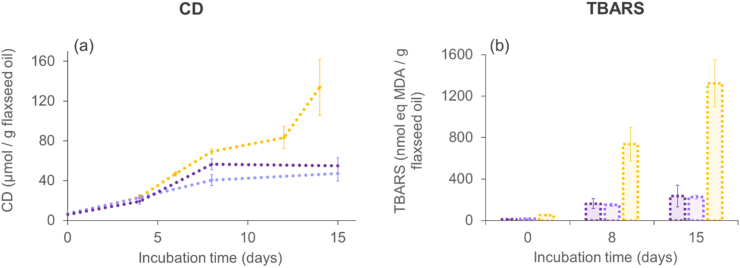
Formation of lipid oxidation products (conjugated dienes (CD), panel (a); and TBARS, panel (b) in a flaxseed oil-based oleofoam prepared with stearyl alcohol/stearic acid (SAL/SAC) in a weight ratio of 7:3 (dark purple) or in the corresponding oleogel (light purple). The yellow symbols/bars correspond to measurements done on pure stripped flaxseed oil (no oleogelator) as a control. Error bars correspond to the standard deviations (n = 4), some of them being within the markers. (For interpretation of the references to colour in this figure legend, the reader is referred to the Web version of this article.)

To confirm the potential protection of highly unsaturated oils by their structuration *via* oleogelation, we also prepared similar systems with stripped flaxseed oil with the mix SAL/SAC. We compared the oxidation kinetics with those of bulk stripped flaxseed oil (Fig. 9). As expected, the reaction occurred faster than with rapeseed oil (therefore, the incubation was conducted over 15 days only). The concentration of lipid oxidation products (CD and TBARS) increased in the oleofoam and in the oleogel over the first 7 days, and then remained fairly stable (reaching around 50 μmol CD/g oil and 230 nmol eq. MDA/g oil, respectively, after 15 days). Conversely, the bulk flaxseed oil showed an increase in the concentration of CD and TBARS over the whole incubation period, reaching eventually much higher values (around 135 μmol CD/g oil and 1300 nmol eq. MDA/g oil, respectively). These results are therefore highly similar to the trends observed with rapeseed oil.

## 4 Discussion

These results show that in both oleofoams and oleogels (with the exception of the GM-based systems), lipid oxidation was initiated within timescales that are fairly aligned with the reaction course occurring in bulk rapeseed oil. However, instead of propagating to reach high oxidation values (as is observed in bulk oil), the reaction was, as a certain point, arrested and the concentration of lipid oxidation products (both for primary and secondary products) remained steady. As lipid oxidation consists of cascaded radical chain reactions (Frankel, 1980; Hwang, 2020; Schaich, 2020b), this suggests that the propagation step of the reaction (leading to an exponentially increasing concentration of lipid radicals and derived hydroperoxides) is, at a certain moment, hampered within the oleofoam and oleogel matrices. Although no data are, to the best of our knowledge, available regarding lipid oxidation in oleofoams, some information has been reported for oleogels (Hwang, 2020). For instance, oleogels based on various PUFA oils and biobased waxes were shown to oxidize less compared to the respective bulk oils (Liu et al., 2020; Yi et al., 2017) which was attributed by the authors to the increased viscosity of the system compared to bulk oil, and hence lower diffusion rates of the reacting species. This effect of oil viscosity on the lipid oxidation kinetics in oil-continuous matrices was previously well-documented by Calligaris et al. (2007), who showed that this parameter is pivotal when developing a mathematical model to describe the temperature dependence of the lipid oxidation rate in such systems. Nevertheless, it is quite remarkable that in our oleofoams and oleogels (with the exception of the GM-based systems), the onset of the formation of lipid oxidation products was very similar to what occurred in bulk oil (or even started slightly earlier, in the case of oleofoams), yet seemed to be arrested at a certain point. A possible explanation is that the radical chain propagation started in multiple locations within the system and proceeded locally within the liquid oil domains (according to the SFC measurements, at 25 °C, only 5.5–9 % of the whole oleogel matrix is solid; Fig. 6), yet was physically prevented to spread further by the crystalline network formed by the oleogelator. Such an effect would be consistent with other studies in the literature showing that long-chain PUFAs oxidized – proportionally – less when diluted in a high melting point oil blend (Hoppenreijs et al., 2021). To the best of our knowledge, such an “arrested propagation” of lipid oxidation in oil-continuous systems has not been reported elsewhere, but an analogy could be made with the findings of Shchepinov and co-workers, who demonstrated in various systems, such as lipid bilayers, that a small proportion of deuterated PUFAs (which cannot be subjected to H abstraction when the bis-allylic groups are deuterated) is able to limit, or even totally block the oxidation of regular PUFAs (Firsov et al., 2019; Hill et al., 2012). This outcome was attributed to the potential of such deuterated PUFAs to interrupt the lipid oxidation radical chain reactions. Although our systems are fundamentally very different, the presence of oleogelator-based structures seems to also yield such a propagation interruption effect. It would be highly interesting, in future work, to investigate further such phenomena and how they may be linked to the structure of the crystalline lipid network.

The case of GM as an oleogelator is also an interesting point to discuss. As mentioned earlier, this component led to oleofams and oleogels that were much more stable to lipid oxidation in comparison with the systems made with the other oleogelators. In fact, no formation of primary nor secondary oxidation products could be detected in the GM-based samples over the whole incubation period (Fig. 8). This was also the case when an additional test of flaxseed oil-GM oleogel was conducted for 15 days (Supplementary information, Fig. S3). We checked whether commercial GM may have contained endogenous antioxidants, such as tocopherols, but this was not the case (Supplementary information, Fig. S1). Therefore, one may assume that the excellent oxidative stability of GM-based systems may be due to specific structural properties. As described in the previous sections, the main difference among the oleofoams is the lower overrun for the GM-based one. We can therefore not exclude that the low oxidation could be associated with the low fraction of air incorporated in comparison to the other oleofoam systems. To wrap up, although more investigations would still be needed to fully unravel the respective effects of the different oleogelators regarding the prevention of lipid oxidation, it seems that the ability to form thin, elongated crystals that confer a high physical stability to the system could be of importance. It is interesting to notice that this criterion is not necessarily linked to physical properties such as SFC or *T*_m_, as they were clearly not discriminating here.

Finally, a last point interesting to take a closer look at is the difference between the oxidizability of a given oleofoam and that of the corresponding oleogel (i.e., made with the same oleogelator). In general, oleofoams oxidized slightly earlier and/or to a higher extent compared to their counterpart oleogel. This is particularly visible for the rapeseed oil-based oleofoams with SAL, SAC, or the mixture thereof (Fig. 8), for which CD formation was even detected slightly earlier than in bulk oil. To check whether this effect was due to the presence of air bubbles in the matrix, or to the initial high-temperature whipping process, an additional control experiment was done where we prepared oleofoams based on rapeseed oil and SAL or the SAL/SAC mixture, which were subsequently re-melted and cooled. In that way, the samples had been subjected to the whipping process, but did not contain air bubbles anymore. Upon incubation, we observed that these two “re-melted oleofoams” also showed a quite early formation of CDs, exactly (or even a bit earlier) as in the regular oleofoams, and then showed oxidation patterns that were very similar (Supplementary info, Fig. S4). This suggests that the initial high-temperature whipping step may be slightly detrimental to the subsequent oxidative stability of the systems, which can be a point of attention for the formulation of such systems targeted to applications.

## 5 Conclusions

In this work, we investigated the oxidative stability of oleofoams prepared from vegetable oils rich in polyunsaturated fatty acids. Various oleogelators were selected based on previous findings, and the physical characterization of the obtained systems was consistent with the current knowledge and design rules for such systems. For instance, the interest of combining stearic acid and stearyl alcohol (SAC and SAL, respectively) as compared to using each component individually was confirmed, and underpinned by an extensive physical and morphological characterization of the systems. To the best of our knowledge, the oxidative stability of oleofoams had not been investigated before, and we showed that these systems hold an interesting potential for limiting the propagation of this radical chain reaction, compared to bulk oil. Glyceryl monostearate-based oleofoams, in particular, were remarkably stable to oxidation, which might be related to the fine lipid crystal needles formed in this system, and to the lower overrun compared to that of the other oleofoams. The probable role of the lipid crystal structure and organization within the oil phase on the oxidative fate of the systems was confirmed by experiments conducted on oleogels, having the same composition as oleofoams but not containing air bubbles, which highlighted that oleogels show fairly similar oxidation patterns as their corresponding oleofoams. A nuance between these two systems may still be found, which could be related to a pro-oxidant effect of the processing route for oleofoams involving high temperature and whipping. This could be a point of attention for forthcoming work aiming at developing PUFA-rich oleofoams for food or other biocompatible applications.

## CRediT authorship contribution statement

**Lucie Ribourg-Birault:** Conceptualization, Data curation, Formal analysis, Investigation, Methodology, Validation, Visualization, Writing – original draft, Writing – review & editing. **Anne Meynier:** Conceptualization, Methodology, Validation, Writing – review & editing, Supervision, Project administration. **Simon Vergé:** Investigation, Validation, Data curation, Visualization, Writing – original draft. **Emeline Sallan:** Investigation, Validation, Data curation, Writing – original draft. **Alice Kermarrec:** Investigation, Validation, Data curation, Writing – review & editing. **Xavier Falourd:** Investigation, Methodology, Validation, Data curation, Visualization, Writing – review & editing. **Claire Berton-Carabin:** Methodology, Validation, Writing – original draft, Writing – review & editing, Supervision, Project administration. **Anne-Laure Fameau:** Conceptualization, Methodology, Validation, Writing – original draft, Writing – review & editing, Supervision, Project administration.

## Declaration of competing interest

The authors declare that they have no known competing financial interests or personal relationships that could have appeared to influence the work reported in this paper.

## Data Availability

Data will be made available on request.
