# Supplementary material for: Oleofoams: The impact of formulating air-in-oil systems from a lipid oxidation perspective
Source: Curr Res Food Sci. 2024 Jan 29;8:100690. doi: 10.1016/j.crfs.2024.100690 (PMC10847802; doi:10.1016/j.crfs.2024.100690)
Supplement: Multimedia component 1 [file mmc1.docx]

**Supporting Information**

**Oleofoams: the impact of formulating air-in-oil systems from a lipid oxidation perspective**

Lucie Ribourg-Birault^a^, Anne Meynier^a^, Simon Vergé^a^, Emeline Sallan^a^, Alice Kermarrec^a^, Xavier Falourd ^a, b^, Claire Berton-Carabin^a,c*^, Anne-Laure Fameau^d*^

^a^ INRAE, UR BIA, F-44300 Nantes, France

^b^ INRAE, PROBE/CALIS Research Infrastructures, BIBS Facility, F-44300, Nantes, France

^c^ Wageningen University & Research, Laboratory of Food Process Engineering, 6700 AA Wageningen, NL

^d^ Univ. Lille, CNRS, INRAE, Centrale Lille, UMET, F-59000 Lille, France

* Corresponding authors: Anne-Laure Fameau ([anne-laure.fameau@inrae.fr](mailto:anne-laure.fameau@inrae.fr)); Claire Berton-Carabin ([claire.berton-carabin@inrae.fr](mailto:claire.berton-carabin@inrae.fr))

**Table S1:** Tocopherol content in rapeseed oil-based oleogels prepared with glyceryl monostearate (GM, light orange), stearyl alcohol (SAL, light blue) and stearic acid (SAC, light red).

**Table S2:** Fatty acid content (g/100g of FA) of stripped rapeseed and flaxseed oil and of glyceryl monostearate (GM) and stearic acid (SAC).

**Table S3:** Influence of recycling delay on the calculated SFC.

**Table S4:** Calculation of the maximum amount of hydroperoxides that could theoretically form in our experimental conditions.

**Figure S1:** Tocopherol content in rapeseed and flaxseed oil, before and after stripping. The error bars correspond to the standard deviation (n=3).

**Figure S2:** Photo of rapeseed oil-based oleofoams produced at 80 °C with glyceryl monostearate (GM, orange), stearic acid (SAC, red), stearyl alcohol (SAL, blue), or stearyl alcohol/stearic acid (SAL/SAC) in a weight ratio of 7:3 (purple).

**Figure S3:** Formation of conjugated dienes (CD) in flaxseed oil-based oleogels prepared with glyceryl monostearate (GM, light orange) or stearyl alcohol/stearic acid (SAL/SAC) in a weight ratio of 7:3 (light purple). The yellow curve corresponds to measurements done on pure stripped flaxseed oil (no oleogelator) as a control. Error bars correspond to the standard deviations (n= 4), some of them being within the markers. The yellow and purple curves have already been presented in Figure 9.

**Figure S4:** Formation of lipid oxidation products: (a) CD and (b) TBARS, in a rapeseed oil-based oleofoam prepared with stearyl alcohol/stearic acid (SAL/SAC) in a weight ratio of 7:3 (dark purple) and with stearyl alcohol (SAL, dark blue) or in the corresponding oleogel (light purple and light blue). The dotted curves/bars correspond to the remelted rapeseed oil-based oleofoams prepared with stearyl alcohol/stearic acid (SAL/SAC) in a weight ratio of 7:3 (dark purple) and with stearyl alcohol (SAL, dark blue). Error bars correspond to the standard deviations (n= 4), some of them being within the markers. *Note: The remelted foams were prepared by reheating the foams produced for 5 minutes in a water bath at 75 °C with magnetic stirring. After returning to room temperature, tubes were placed in an ice bath for 10 minutes.*
